# Effects of simultaneous cognitive and aerobic exercise training on dual-task walking performance in healthy older adults: results from a pilot randomized controlled trial

**DOI:** 10.1186/s12877-020-1484-5

**Published:** 2020-03-02

**Authors:** David A. Raichlen, Pradyumna K. Bharadwaj, Lauren A. Nguyen, Mary Kathryn Franchetti, Erika K. Zigman, Abigail R. Solorio, Gene E. Alexander

**Affiliations:** 1 Human and Evolutionary Biology Section, Department of Biological Sciences, University of Southern California, 3616 Trousdale Parkway, Los Angeles, CA 90089-0372 USA; 2 Department of Psychology, University of Arizona, 1503 E. University, Tucson, AZ 85721 USA; 3 Evelyn F. McKnight Brain Institute, University of Arizona, Tucson, AZ USA; 4 Department of Psychiatry, University of Arizona, Tucson, AZ USA; 5 Neuroscience Graduate Interdisciplinary Program, University of Arizona, Tucson, AZ USA; 6 Physiological Sciences Graduate Interdisciplinary Program, University of Arizona, Tucson, AZ USA; 7 BIO5 Institute, University of Arizona, Tucson, AZ USA; 8 Arizona Alzheimer’s Consortium, Phoenix, AZ USA

**Keywords:** Physical activity, Cognition, Aging, Exergame, Executive function

## Abstract

**Background:**

The ability to walk and perform cognitive tasks simultaneously is a key aspect of daily life. Performance declines in these dual-tasks may be associated with early signs of neurodegenerative disease and increased risk of falls. Thus, interventions to improve dual-task walking performance are of great interest for promoting healthy aging. Here, we present results of a pilot randomized controlled trial (RCT) to evaluate the effects of a simultaneous aerobic exercise and cognitive training intervention on dual-task walking performance in healthy older adults.

**Methods:**

Community-dwelling, healthy older adults were recruited to participate in a 12-week RCT. Participants were randomized into one of four groups (*n* = 74): 1) cognitive training (COG), 2) aerobic exercise (EX), 3) combined aerobic exercise and cognitive training (EXCOG), and 4) video-watching control (CON). The COG and EXCOG groups both used a tablet-based cognitive training program that challenged aspects of executive cognitive function, memory, and processing speed. Performance on a dual-task walking test (DTWT; serial subtraction during two-minute walk) was assessed by researchers blinded to groupings before the intervention, and at 6 and 12 weeks. We included all participants randomized with baseline measurements in an intention to treat analysis using linear mixed effects models.

**Results:**

We found a significant group by time interaction for cognitive performance on the DTWT (*p* = 0.039). Specifically, participants in the EXCOG, EX, and COG groups significantly improved on the cognitive aspect of the DTWT following the full 12-week intervention (*p* = 3.5e-7, *p* = 0.048, *p* = 0.048, respectively). The improvements in EXCOG were twice as large as in the other groups, and were significant at 6 weeks (*p* = 0.019). The CON group did not show a significant change in cognitive performance on the DTWT, and no group significantly altered dual-task gait measures following the intervention.

**Conclusions:**

A simultaneous aerobic exercise and cognitive training intervention significantly improved cognitive performance during a DTWT in healthy older adults. Despite no change in DTWT gait measures, significant improvements in cognitive performance indicate that further investigation in a larger RCT is warranted.

**Trial registration:**

Clinicaltrials.gov, NCT04120792, Retrospectively Registered 08 October 2019.

## Background

Performance on tasks that require executive attention and dual control of motor and cognitive functions tend to decline with age [1, 2], and this decline is exacerbated in neurodegenerative conditions including Parkinson’s disease and Alzheimer’s disease (AD) [3, 4]. In fact, poor dual-task performance may be an early indicator of dementia and can greatly impact activities of daily life [3]. Individuals who perform poorly on dual-task tests (e.g., walking while engaged in cognitively demanding tasks) are also likely to have balance problems and are at increased risk for falls [4]. Thus, finding ways to improve dual-task ability may reduce functional decline and mediate risk of falls, especially in individuals experiencing age-related cognitive decline [4].

Recent work generally suggests exercise training, and combining cognitive and motor challenges in long-term interventions may improve both gait and cognitive performance in dual-task tests [5–7]. However, results are not always consistent, and several studies have found that exercise interventions alone, or combined exercise and cognitive activity interventions did not significantly improve performance on dual-task walking tests [8–12]. Additionally, in those studies that report beneficial outcomes, it is unclear whether the effects are due mainly to the aerobic exercise component, the cognitive aspects of training, or the combined benefits of both [7], and there is limited evidence of transfer effects from one dual-task paradigm to another [8]. Finally, combined cognitive and exercise training often requires specialized equipment (e.g., stationary exercise equipment and/or technology to implement the cognitive component; see [13–16]), which may be an impediment to widespread use of such training paradigms. Thus, while combined interventions have shown promise, there is a need for RCTs that are able to evaluate the effects of the physical exercise and cognitive aspects of training programs so that effective interventions can be most efficiently implemented.

To investigate the relative contributions of exercise and cognitive training on dual-task performance in older adults, we developed an intervention that simultaneously challenges aerobic and cognitive activity. We use moderate intensity aerobic exercise in this intervention, as this form of physical activity has shown the most consistent beneficial effects on cognitive outcomes in older adults [17] and more easily accommodates a simultaneous cognitive stimulus presented on a tablet computer. As a proof of concept, we have tested this intervention in a small sample of healthy older adults, forming the foundation for larger RCTs. Here, we present results of this pilot RCT examining the effects of a simultaneous aerobic and cognitive training intervention on dual-task walking performance compared with exercise alone, cognitive training alone, and a video-watching control group.

## Methods

### Participants

Participants were community-dwelling, generally healthy older adults recruited by advertising in newsletters and newspapers from the local Tucson-metro area from May 2015 through August 2017, and were screened for health status, cognitive impairment, and current physical activity levels. We included only individuals aged 60–74 who participated in less than 1 hour per week of aerobic exercise. Sample size was calculated by an a priori power analysis to determine the number of participants needed per group, assuming a medium effect size with 80% power (*p* < 0.05 two-tailed). Participants were considered eligible to participate in the study if they reported no significant concerns about their memory or other cognitive abilities and they had no history of a major neurological, psychiatric, or medical disorder or injury that would affect cognitive or physical function (including stroke, heart disease, neurodegenerative disease, sleep apnea, substance use or abuse, uncontrolled depression, psychosis or other psychiatric disorder, uncontrolled hypertension, brain cancer, significant head injury with loss of consciousness, peripheral injury or significant arthritis). All participants had a score ≥ 26 on the Mini-Mental State Exam (MMSE [18]) and were required to provide a written statement from their primary care physician indicating that they were able to participate in the 12-week exercise program and did not have any medical conditions or physical limitations that would affect their ability to participate in this study of healthy older adults (see Supplementary Table S1 for medication usage). Individuals who met the inclusion criteria provided their informed written consent to participate in this study and were randomized into one of four groups (see below). This study adheres to the CONSORT guidelines for reporting results from clinical trials. All procedures were approved by the University of Arizona Institutional Review Board.

### Interventions

Participants were randomly assigned to one of four parallel interventions: 1) cognitive training (COG), 2) aerobic exercise (EX), 3) combined aerobic exercise and cognitive training (EXCOG), 4) video-watching control (CON). Each of these interventions is explained in more detail below. Randomization was determined using a computerized random number generator by a research coordinator who was not involved in enrollment or assessment. Participants did not learn of their allocation until the first day of the intervention. All interventions lasted 12 weeks, and participants came to the trial facilities at the University of Arizona 3 days per week. All sessions for all groups took place in a suite of four identical testing rooms. Participants completed each session with only a research assistant present in the room.

#### Cognitive training (COG)

Participants assigned to the COG group engaged in cognitive training 3 days per week. The cognitive training activity was designed to challenge multiple cognitive domains across a 30-min period, including memory, executive functions, and processing speed. The overall task was to navigate in a tablet-based maze, controlling movement along roads from starting to ending locations using hand-held controllers. The roads traveled within the maze included observable objects placed periodically on the landscape (near road T-junctions) to provide background visual cues. At the beginning of a session, a maze was randomly generated, and the participant was able to use an aerial view map to navigate through the maze using custom designed handheld controllers. The controllers allowed turns based on differential right/left hand squeeze pressure and the participant’s current location within the maze was indicated by a blue triangle whose position was updated on the maze map in real time during the task. When participants completed the maze, they were transported back to the starting location and continued to perform the same maze task repeatedly until the maze learning portion of the session was completed.

While navigating through the maze task, a series of additional cognitive tasks were presented using two placards that appear on the tablet screen on either side of the road (see Supplementary Figures S1 and S2). The order of the tasks presented were: 1) verbal paired-associates - learning condition, 2) Simon inhibition task, 3) letter/number switching, 4) N-back, 5) simple and choice reaction time, and 6) verbal paired-associates - memory condition. These six additional tasks were presented for 2–3 min each. For the verbal paired-associates - learning condition, a series of 25 object word-pairs (randomly selected from a pool of 275 for each session) were presented, one word on each of the two placards; and participants were asked to press the right/left handheld buttons if both objects in the pairs were living/non-living. The Simon task testing inhibition of prepotent responses [19] randomly presented a series of arrows on either the right or left placard, which pointed either in the right or left direction; and participants pressed the right or left handheld buttons corresponding to the direction pointed by each arrow. For the letter/number switching task (adapted from Rogers & Monsell [20]), participants were presented with single letter-number pairs either in the top half, bottom half, or switching between top and bottom halves of one of two randomly selected placards; and participants pressed the right/left handheld buttons if the number was even/odd when presented on the top half or if the letter was a vowel/consonant when presented on the bottom half. The N-back task presented a series of single-digits on one of two randomly selected placards; and participants were asked to press the right-hand button when a number equaled a digit displayed 2-back from the currently displayed number. The simple and choice reaction time tasks first presented the simple condition with an ‘O’ on either the left or right placard and participants pressed both buttons when the target appeared; and for the choice condition, participants were presented with single letters on the left and right placards and were asked to press the right/left buttons when the letters were the same/different. The verbal paired-associates delay condition presented 15 word-pairs, including 10 that were previously presented earlier in the session; and participants were asked to press the right/left buttons if the pair was/was not previously presented. For the last 4 min of the cognitive training session, the aerial view map of the maze was removed from the screen and participants were asked to then only navigate the maze relying on their spatial memory of visual cues observed in the landscape.

#### Exercise training (EX)

Participants in the EX group engaged in aerobic exercise training on a stationary recumbent bicycle (XBR95, Spirit Fitness, Jonesboro, AR) three times per week for 12 weeks. Over the first 2 weeks, participants gradually increased the target intensity and time spent at target intensity until they achieved the goal aerobic session for the final 10 weeks. During the first week of training, participants were asked to cycle at 40% of Heart Rate Reserve (HRR) for 15 min. HRR is calculated using heart rate (HR) at rest and age-adjusted estimated maximum heart rate (from [21]) as: [HR_max_ – HR_rest_] (percentage values of HRR calculated as: %HRR*[HR_max_ – HR_rest_] + HR_rest_). In the second week, participants were asked to cycle at 40–50% of HRR for 20 min. Beginning in week 3, participants were asked to cycle at 50–80% of their heart rate reserve (HRR) for 30 min, preceded and followed by 5 min of cycling at less than 50% of HRR. Heart rate was measured using a chest strap monitor (Polar H7 Heart Rate Sensor, Polar Electro Oy, Finland), and exercise cycle resistance or pedal revolutions per minute were changed to maintain target heart rates both during and across exercise training sessions by research study staff.

#### Combined aerobic exercise and cognitive training (EXCOG)

Participants in the EXCOG group engaged in simultaneous exercise and cognitive training three times per week for 12 weeks. This intervention combined the EX and COG conditions described above. Participants engaged in the same aerobic exercise training regimen as the EX group, however, while cycling at the prescribed intensity, participants simultaneously engaged in the cognitive training tasks. The order and length of tasks was the same as described above for the COG group.

#### Video-watching control (CON)

Participants in the CON group engaged in a neutral cognitive activity three times per week for 12 weeks. This activity involved watching videos on a tablet computer for 30 min. Videos were chosen to be neutral for interest and content (e.g., nature documentaries).

### Dual-task measurement procedure

On three occasions (baseline, intervention mid-point [6-week], and post-intervention [12-week]), participants completed, in a counterbalanced order, a single-task and dual-task walk along a 15.25 m course in an enclosed hallway. This task differs from the cycling-based multi-tasking that occurs during the intervention, allowing us to assess the potential for transfer effects. During these tasks, wearable accelerometers (Biosensics, Watertown, MA) were affixed to the left and right lower leg just below the knee to measure spatiotemporal kinematics. The course was marked by cones at either end and participants were asked to walk comfortably at a normal walking pace for 2 min. During the single-task condition, participants walked as far as they could at this pace, and the distance walked was measured. During the dual-task condition, subjects were given the same instruction, but were also asked to serially subtract 7’s beginning at 500. Their answers were recorded for analysis of both speed and accuracy. To exclude gait data from turns at the ends of the hallway, we applied a median filter and removed strides where stride velocity was greater or less than 2 SDs from the median (see [22]). In addition, we excluded the first 10 strides to account for initial acceleration to a comfortable walking pace. The primary outcome for this study was cognitive performance during the dual-task test. Secondary outcomes for this study were gait parameters during dual-task walking. Gait parameters analyzed were: stride time, stride length, and stride velocity. For each of these variables, we examined mean values and variability (determined by the coefficient of variation). Sample sizes differ between the gait and cognitive variables because equipment issues (loss of Bluetooth signal) led to loss of kinematic data during a small number of trials. Testers were not the same research assistants who performed the interventions and were blinded to participant group membership.

### Data analysis

**Fig. 1:**
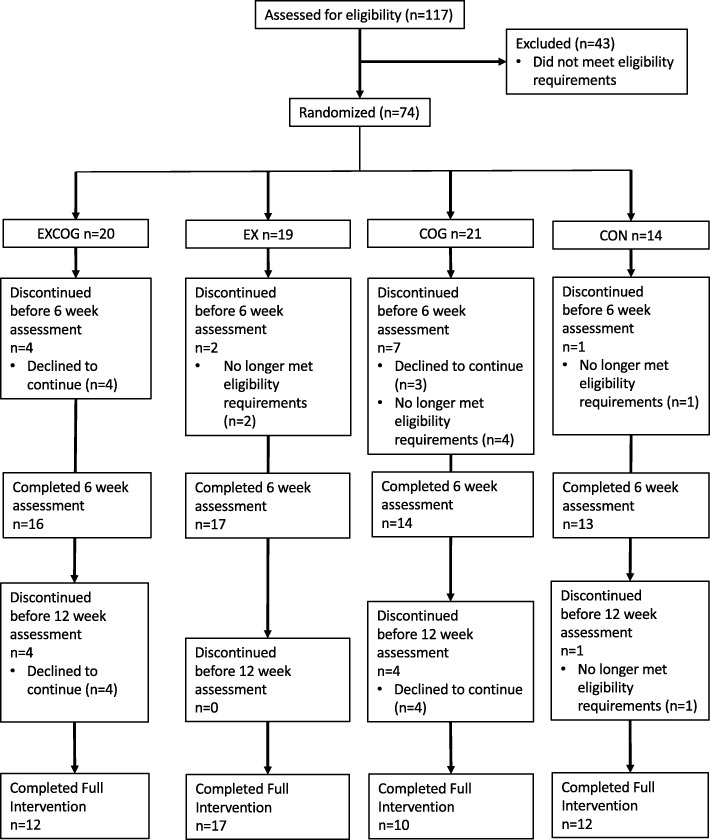
Flow diagram of screening, randomization, intervention, and data analysis

Changes in dual-task performance were assessed at three time points (baseline, 6-week, and 12-week). To compare gait parameters across these time points, we calculated a dual-task cost (DTC) as: [dual task – single task]/[single task] * 100 (following [3]). For cognitive performance, our primary outcome was the total number of correct answers provided during the dual-task challenge. For all outcomes, we compared performance using linear mixed effects models (LMM), with group membership, time point, and the interaction between group and time included in the models as fixed effects, and participant included in the models as random effects. We used false discovery rate (FDR) to control for multiple comparisons. Effect sizes were calculated using post-hoc contrasts from LMMs. For these analyses we included all participants randomized with baseline measurements in an intention to treat analysis (see Fig. 1). LMMs use all available data, including participants with missing data, account for correlations among repeated measurements in individuals, and provide unbiased estimates of effectiveness under missing completely at random (MCAR) and missing at random (MAR) assumptions [23]. LMMs are also a robust analytic method when used in studies with differential participant attrition across intervention arms [24]. All statistical analyses were performed in the R statistical computing environment (R version 3.5.1), using the lme4 and multcomp packages.

## Results

**Fig. 2:**
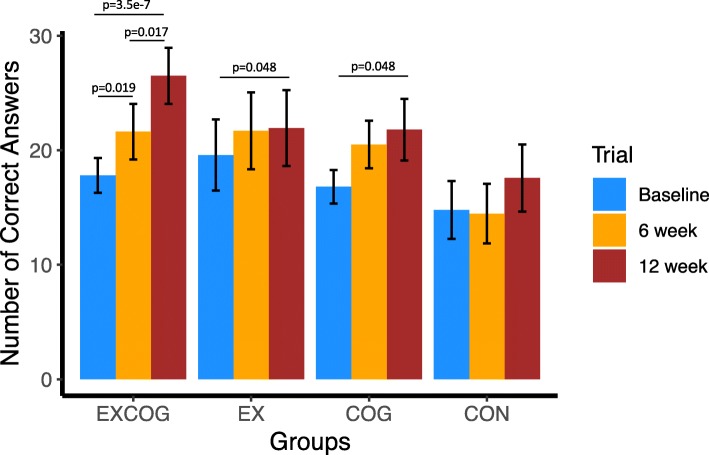
Cognitive performance across time and group. There was a significant interaction between group and time (*p* = 0.039). In post-hoc comparisons, EXCOG (combined exercise and cognitive training) showed significant improvements in the number of correct answers given during the 2-min dual task walking test. EX (exercise only) and COG (cognitive training only) each showed significant, but more modest, improvements only after the full 12-week intervention, CON (video-watching control) showed no significant improvements across any time points. *P*-values shown are corrected for multiple comparisons using FDR, *p* < 0.05. Error bars are SE of the mean

At baseline, individuals in the four intervention groups did not differ significantly in age, gender ratio, body mass index, years of education, or MMSE score (Table 1). All participants who completed either the 6- or 12-week outcome assessments also completed at least 85% of intervention sessions (three participants in EXCOG did not complete one session; one participant in EX did not complete one session; one participant in COG did not complete one session; one participant in EXCOG completed all sessions but did not consistently engage in all aspects of the cognitive tasks in the multi-tasking paradigm during four sessions). We found a significant group by time interaction for cognitive performance on the dual-task test (*p* = 0.039; Supplementary Table S2). Participants in the EXCOG group showed a significant improvement in cognitive performance during the dual-task test for the both 6 and 12 weeks of intervention, after accounting for multiple comparisons (Table 2; Fig. 2; Supplementary Table S3). Effect sizes for post-hoc comparisons in the EXCOG group were medium at 6 weeks (0.47) and large following the 12-week intervention (0.98). Participants in the EX group showed a significant improvement in cognitive performance only after 12 weeks of the intervention (Fig. 2; Table 2; see Supplementary Table S3) with an effect size less than half that of EXCOG (0.39). Participants in the COG group also showed a significant improvement after 12 weeks of the intervention, but an effect size less than half that of EXCOG (0.43). The CON group did not show a significant change on their cognitive performance during the dual-task test across any of the time points. All groups at all time points showed significant dual-task costs in most gait parameters, taking shorter length, longer duration, and lower velocity strides in dual-task compared with single-task conditions (Table 2). However, none of the groups showed a significant change in kinematic dual-task costs across the time points (Table 2; see Supplementary Tables S4-S9).

**Table 1:** Demographic characteristics of participants included in the analysis

Characteristics	EXCOG (*n* = 20)	EX (*n* = 19)	COG (*n* = 21)	CON (*n* = 14)	p
Age, yrs	67.67 (4.65)	68.1 (3.92)	66.35 (3.89)	69.28 (4.34)	0.30
Sex	13 (65)	11 (57.89)	14 (66.67)	9 (64.29)	0.95
Education, yrs	16.90 (2.94)	16.37 (1.95)	17.14 (1.93)	15.71 (2.16)	0.28
BMI, kg/m^2^	28.26 (4.05)	27.98 (4.3)	28.21 (3.46)	29.91 (5.23)	0.59
MMSE	29.00 (1.17)	29.11 (0.94)	28.95 (1.36)	29.21 (1.12)	0.91

^a^*p*-values are given for generalized linear models. Values in table are mean (sd) (values for sex are number of females and percent of females in sample)

**Table 2:** Results of dual-task walking tests across intervention groups and time points

	EXCOG			EX			COG			CON			
	baseline	6-week	12-week	baseline	6-week	12-week	baseline	6-week	12-week	baseline	6-week	12-week	p-val
Cognitive performance													
n	20	16	12	19	17	17	21	14	10	14	13	12	
number correct, mean (SD)	17.8 (6.83)	21.62 (9.69)	26.5 (8.48)	19.58 (13.55)	21.7 (13.83)	21.94 (13.64)	16.82 (6.71)	20.5 (7.78)	21.8 (8.52)	14.79 (9.49)	14.46 (9.4)	17.58 (10.15)	0.039
Gait variables (Dual-task cost, %)													
n	15	11	9	12	11	11	18	9	7	12	11	10	
Stride Length	−4.36 (2.8)	−1.87 (3.02)	−2.35 (1.33)	−4.26 (2.78)	− 1.87 (4.54)	− 1.85 (2.14)	−5.67 (2.27)	−3.96 (3.91)	−3.23 (1.35)	−5.37 (5.21)	−5.07 (3.38)	− 4.73 (4.59)	0.790
Stride Length Variability	0.16 (0.4)	−0.02 (0.09)	0.02 (0.2)	0.09 (0.29)	−0.07 (0.32)	−0.06 (0.19)	0.19 (0.26)	0 (0.22)	0.1 (0.39)	0.1 (0.31)	0.08 (0.16)	0.06 (0.3)	0.891
Stride Duration	4.94 (6.54)	5.35 (7.35)	5.14 (7.5)	8.74 (5.89)	7.89 (6.63)	3.83 (4.75)	8.42 (10.32)	3.88 (3.1)	2.4 (3.22)	10.48 (13.32)	7.48 (9.09)	6.93 (8.98)	0.773
Stride Duration Variability	45 (68.97)	34.32 (72.94)	8.57 (30.82)	43.28 (45.6)	27.16 (54.45)	19.12 (25.56)	40.50 (31.99)	4.68 (18.14)	35.92 (49.66)	40 (46.4)	40.29 (48.2)	37.55 (44.38)	0.439
Stride Velocity	−8.45 (6.86)	−6.36 (7.39)	−6.73 (6.6)	−11.6 (5.75)	−8.36 (6.95)	−5.25 (4.62)	− 12.3 (6.91)	−7.42 (5.67)	− 5.61 (3.48)	−12.86 (12.89)	− 10.8 (9.94)	−10.06 (10.69)	0.925
Stride Velocity Variability	17.91 (21.46)	5.53 (17.77)	0.58 (20.09)	17.96 (34.44)	1.17 (32.26)	0.65 (15.74)	22.92 (22.23)	4.9 (21.78)	4.52 (18.27)	15.82 (33.94)	14.47 (19.1)	13.94 (23.05)	0.739

Note: Data presented are mean (SD); variability is the coefficient of variation for a given variable. Number correct were determined during the dual-task condition only. Gait variables are the dual task cost calculated from both dual and single task trials (see text for further explanation). *P*-values are the interaction of group and time point for each variable from linear mixed effects models

## Discussion

The results of this pilot RCT study suggest that a 12-week intervention that combines multiple cognitive tasks with simultaneous aerobic exercise in healthy older adults can improve cognitive performance during a serial-subtraction dual-task walking test. In fact, in the EXCOG group, participants improved by nearly 50% on the task, and improvements were observed after only 6 weeks of training. The exercise-only (EX) and cognitive training-only (COG) groups also showed improvements in cognitive performance during the dual-task test, however improved performance only reached significance after the complete 12-week intervention, and the size of the improvements were less than half the size in each condition than that found in the EXCOG group. Based on this pilot study, we observed clear benefits for simultaneous aerobic and cognitive training over and above each modality alone. Whether these combined effects are additive or synergistic is unclear and determining how these combined cognitive and physical tasks interact should be a key element of future work. While the EXCOG paradigm was a form of dual-task training, our results show transfer effects to other dual-task paradigms. In the intervention, training occurred on a stationary bicycle and the cognitive challenges were different from those used in the dual-task test that served as our primary outcome, which required serial-subtractions while walking.

Interestingly, there was no effect of intervention group on gait measures across the trial. Previous work has generally shown that cognitively healthy older adults prioritize gait and posture over cognitive performance during challenging dual-task tests [25, 26], and in general, gait parameters are altered under simultaneous cognitive load [4]. Our results suggest that the improved cognitive performance following the EX, COG, and EXCOG interventions were not the result of a change in prioritization between cognitive and motor functions (e.g., increased cognitive performance combined with decreased gait performance). Instead, it appears that EXCOG training, and to a lesser extent EX alone and COG alone, allowed participants to allocate greater resources to cognitive performance without appreciably affecting their gait.

Dual-task tests target attention and executive cognitive functions, domains that are diminished in cognitive aging and often affected early in the course of neurodegenerative disease. Executive cognitive functions can be linked with gait, and walking itself is considered a demanding activity for aspects of attention and executive function [4]. Previous work suggests exercise can improve cognitive function in older adults, with the strongest effects on executive functions and memory [27–33]. Although limited in number, previous studies of simultaneous exercise and cognitive interventions have shown greater effects on executive function after the combined challenge compared with exercise alone [13], or without a control group in patients with mild cognitive impairment [14], and simultaneous training may have a greater effect on executive function compared to cognitive training alone [34]. Our findings extend this work, and by including all relevant control groups, we help to clarify the unique impact of combined interventions. Here, combined simultaneous exercise and cognitive training outperforms exercise or cognitive training alone in improving cognitive performance during a dual-task requiring aspects of divided attention, working memory, and executive control of cognitive and motor functions.

While our results demonstrate the potential advantages of the EX, COG, and EXCOG paradigms for improving cognitive performance during walking, our study does have some limitations that point to directions for future work. First, our sample size in this pilot study is small, limiting our overall power, and studies with larger samples are needed to determine the generalizability of our results. Second, a limitation of this study was the high dropout rate and future clinical trials of this intervention may consider making it easier for participants to continue in the program. The challenge of the cognitive training within the EXCOG and COG conditions may have led to greater drop-out during the intervention. While it is possible that differential attrition may have increased the possibility of bias in the results, EXCOG outperformed COG on the dual-task test, suggesting the benefits of the simultaneous intervention are not attributable to potential sample differences in cognitive training engagement. However, future studies should examine the difficulty of the cognitive tasks to determine whether reductions in cognitive challenges can help to reduce attrition in these groups while maintaining beneficial outcomes. Future work should also include qualitative assessments of the acceptability of the cognitive challenges to help determine whether specific aspects of the tasks were associated with individual drop-out. Third, we did not collect brain imaging scans as part of this RCT and cannot address aspects of brain structure, function, and connectivity that may be associated with our observed dual-task cognitive differences in interventions. Determining brain structural, functional, and connectivity changes that underlie improvements in dual-task performance in response to simultaneous versus individual modality training sessions will help us understand which interventions have the greatest potential to offset the impact of brain aging and developing neurodegenerative diseases, like AD. Finally, future trials of simultaneous exercise and cognitive challenge interventions should address effects on changes in other aspects of cognition and should examine different exercise intensities and modalities (including the addition of resistance training) to help clarify the most effective prescription for improving cognitive performance during aging.

## Conclusions

Our results show that EXCOG interventions may have a unique benefit, over and above exercise or cognitive training alone, on the ability of individuals to engage in cognitively demanding activities during dual-task motor activities. The findings suggest that future work should include larger samples and should focus on determining the nature of the interaction between aerobic and cognitive challenges during combined interventions. If they are indeed additive, this result would suggest that we may be able to alter the intensity of the challenges in ways that can be tailored for individuals with different cognitive and physical activity needs. If, on the other hand, the combination of aerobic and cognitive challenges has synergistic effects, efforts should be made to determine the ideal levels of interactive challenge or difficulty for each activity to maximize the cognitive and functional benefits. Based on the results of this pilot study, we believe future work should focus on a larger clinical trial of this intervention in healthy populations, followed by studies in populations at increased risk for neurodegenerative disease since performance on dual-task tests may be a potential early indicator of dementia [3].

## Supplementary information

**Additional file 1: Table S1.** Medication usage across groups. **Table S2.** Linear mixed effects model of cognitive function during dual task for individuals who completed the 6 week test. **Table S3.** Post-hoc contrasts for Linear mixed effects model of cognitive function during dual task for individuals who completed the 6 week test. **Table S4.** Linear mixed effects model of dual task cost on stride length. **Table S5.** Linear mixed effects model of dual task cost on CV of stride length. **Table S6** Linear mixed effects model of dual task cost on stride duration. **Table S7.** Linear mixed effects model of dual task cost on CV of stride duration. **Table S8.** Linear mixed effects model of dual task cost on stride velocity. **Table S9.** Linear mixed effects model of dual task cost on CV of stride velocity. **Figure S1.** View of maze and road as seen by participants. **Figure S2.** Task example.

## Data Availability

The dataset used during this study are available from the corresponding author upon reasonable request.

## Abbreviations

AD: Alzheimer’s disease
COG: Cognitive training only group
CON: Control group
EX: Exercise training only group
EXCOG: Simultaneous cognitive and exercise training group
HRR: Heart Rate Reserve
LMM: Linear mixed effects model
MMSE: Mini-mental state exam
RCT: Randomized controlled trial

## References

[1] Alexander GE, Ryan L, Bowers D, Foster TC, Bizon JL, Geldmacher DS, Glisky EL (2012). Characterizing cognitive aging in humans with links to animal models. Front Aging Neurosci.

[2] Park DC, Reuter-Lorenz P (2009). The adaptive brain: aging and neurocognitive scaffolding. Annu Rev Psychol.

[3] Schwenk M, Zieschang T, Oster P, Hauer K (2010). Dual-task performances can be improved in patients with dementia: a randomized controlled trial. Neurology.

[4] Yogev-Seligmann G, Hausdorff JM, Giladi N (2008). The role of executive function and attention in gait. Mov Disord.

[5] Bruderer-Hofstetter M, Rausch-Osthoff A-K, Meichtry A, Münzer T, Niedermann K (2018). Effective multicomponent interventions in comparison to active control and no interventions on physical capacity, cognitive function and instrumental activities of daily living in elderly people with and without mild impaired cognition–a systematic review and network meta-analysis. Ageing Res Rev.

[6] Plummer P, Zukowski LA, Giuliani C, Hall AM, Zurakowski D (2016). Effects of physical exercise interventions on gait-related dual-task interference in older adults: a systematic review and meta-analysis. Gerontology.

[7] Raichlen DA, Alexander GE (2017). Adaptive capacity: an evolutionary neuroscience model linking exercise, cognition, and brain health. Trends Neurosci.

[8] Fraser SA, Li KZ-H, Berryman N, Desjardins-Crépeau L, Lussier M, Vadaga K, Lehr L, Vu M, Tuong T, Bosquet L (2017). Does combined physical and cognitive training improve dual-task balance and gait outcomes in sedentary older adults?. Front Hum Neurosci.

[9] Gobbo S, Bergamin M, Sieverdes JC, Ermolao A, Zaccaria M (2014). Effects of exercise on dual-task ability and balance in older adults: a systematic review. Arch Gerontol Geriatr.

[10] Makizako H, Doi T, Shimada H, Yoshida D, Tsutsumimoto K, Uemura K, Suzuki T (2012). Does a multicomponent exercise program improve dual-task performance in amnestic mild cognitive impairment? A randomized controlled trial. Aging Clin Exp Res.

[11] Plummer-D'Amato P, Cohen Z, Daee NA, Lawson SE, Lizotte MR, Padilla A (2012). Effects of once weekly dual-task training in older adults: a pilot randomized controlled trial. Geriatr Gerontol Int.

[12] Wollesen B, Voelcker-Rehage C (2014). Training effects on motor–cognitive dual-task performance in older adults. Eur Rev Aging Phys Act.

[13] Anderson-Hanley C, Arciero PJ, Brickman AM, Nimon JP, Okuma N, Westen SC, Merz ME, Pence BD, Woods JA, Kramer AF (2012). Exergaming and older adult cognition: a cluster randomized clinical trial. Am J Prev Med.

[14] Anderson-Hanley C, Barcelos NM, Zimmerman EA, Gillen RW, Dunnam M, Cohen BD, Yerokhin V, Miller KE, Hayes DJ, Arciero PJ (2018). The aerobic and cognitive exercise study (ACES) for community-dwelling older adults with or at-risk for mild cognitive impairment (MCI): neuropsychological, neurobiological and neuroimaging outcomes of a randomized clinical trial. Front Aging Neurosci.

[15] Anderson-Hanley C, Stark J, Wall KM, VanBrakle M, Michel M, Maloney M, Barcelos N, Striegnitz K, Cohen BD, Kramer AF (2018). The interactive physical and cognitive exercise system (iPACes™): effects of a 3-month in-home pilot clinical trial for mild cognitive impairment and caregivers. Clin Interv Aging.

[16] Barcelos N, Shah N, Cohen K, Hogan MJ, Mulkerrin E, Arciero PJ, Cohen BD, Kramer AF, Anderson-Hanley C (2015). Aerobic and cognitive exercise (ACE) pilot study for older adults: executive function improves with cognitive challenge while exergaming. J Int Neuropsychol Soc.

[17] Northey JM, Cherbuin N, Pumpa KL, Smee DJ, Rattray B (2018). Exercise interventions for cognitive function in adults older than 50: a systematic review with meta-analysis. Br J Sports Med.

[18] Folstein MF, Folstein SE, Mchugh PR (1975). “Mini-mental state”: a practical method for grading the cognitive state of patients for the clinician. J Psychiatr Res.

[19] Simon JR. The effects of an irrelevant directional cue on human information processing. In: Proctor RW, Reeves TG, editors. Advances in psychology. Stimulus-response compatibility: An integrated perspective. North-Holland, Oxford; 1990. p. 31–86.

[20] Rogers RD, Monsell S (1995). Costs of a predictible switch between simple cognitive tasks. J Exp Psychol Gen.

[21] Tanaka H, Monahan KD, Seals DR (2001). Age-predicted maximal heart rate revisited. J Am Coll Cardiol.

[22] Hausdorff JM, Rios DA, Edelberg HK (2001). Gait variability and fall risk in community-living older adults: a 1-year prospective study. Arch Phys Med Rehabil.

[23] Gueorguieva R, Krystal JH (2004). Move over anova: progress in analyzing repeated-measures data andits reflection in papers published in the archives of general psychiatry. Arch Gen Psychiatry.

[24] Bell ML, Kenward MG, Fairclough DL, Horton NJ (2013). Differential dropout and bias in randomised controlled trials: when it matters and when it may not. BMJ.

[25] Bloem BR, Valkenburg VV, Slabbekoorn M, Willemsen MD (2001). The multiple tasks test: development and normal strategies. Gait Posture.

[26] Simoni D, Rubbieri G, Baccini M, Rinaldi L, Becheri D, Forconi T, Mossello E, Zanieri S, Marchionni N, Di Bari M (2013). Different motor tasks impact differently on cognitive performance of older persons during dual task tests. Clin Biomech.

[27] Anderson-Hanley C, Nimon JP, Westen SC (2010). Cognitive health benefits of strengthening exercise for community-dwelling older adults. J Clin Exp Neuropsychol.

[28] Best JR, Nagamatsu LS, Liu-Ambrose T (2014). Improvements to executive function during exercise training predict maintenance of physical activity over the following year. Front Hum Neurosci.

[29] Colcombe S, Kramer AF (2003). Fitness effects on the cognitive function of older adults: a meta-analytic study. Psychol Sci.

[30] Erickson KI, Voss MW, Prakash RS, Basak C, Szabo A, Chaddock L, Kim JS, Heo S, Alves H, White SM (2011). Exercise training increases size of hippocampus and improves memory. Proc Natl Acad Sci.

[31] Etnier JL, Chang Y-K (2009). The effect of physical activity on executive function: a brief commentary on definitions, measurement issues, and the current state of the literature. J Sport Exer Psychol.

[32] Hillman CH, Belopolsky AV, Snook EM, Kramer AF, McAuley E (2004). Physical activity and executive control: implications for increased cognitive health during older adulthood. Res Q Exerc Sport.

[33] Roig M, Nordbrandt S, Geertsen SS, Nielsen JB (2013). The effects of cardiovascular exercise on human memory: a review with meta-analysis. Neurosci Biobehav Rev.

[34] Theill N, Schumacher V, Adelsberger R, Martin M, Jäncke L (2013). Effects of simultaneously performed cognitive and physical training in older adults. BMC Neurosci.

