# Supplementary material for: Effects of simultaneous cognitive and aerobic exercise training on dual-task walking performance in healthy older adults: results from a pilot randomized controlled trial
Source: BMC Geriatr. 2020 Mar 2;20:83. doi: 10.1186/s12877-020-1484-5 (PMC7052975; doi:10.1186/s12877-020-1484-5)
Supplement: Supplementary file 1 — Additional file 1: Table S1. Medication usage across groups. Table S2. Linear mixed effects model of cognitive function during dual task for individuals who completed the 6 week test. Table S3. Post-hoc contrasts for Linear mixed effects model of cognitive function during dual task for individuals who completed the 6 week test. Table S4. Linear mixed effects model of dual task cost on stride length. Table S5. Linear mixed effects model of dual task cost on CV of stride length. Table S6 Linear mixed effects model of dual task cost on stride duration. Table S7. Linear mixed effects model of dual task cost on CV of stride duration. Table S8. Linear mixed effects model of dual task cost on stride velocity. Table S9. Linear mixed effects model of dual task cost on CV of stride velocity. Figure S1. View of maze and road as seen by participants. Figure S2. Task example. [file 12877_2020_1484_MOESM1_ESM.docx]

**SUPPLEMENARY MATERIALS**

SUPPLEMENTARY TABLES

Table S1. Medication usage across groups.

| **Medication Category** | **EXCOG (n=20)** | **EX (n=19)** | **COG (n=21)** | **CON (n=14)** | **p** |
| --- | --- | --- | --- | --- | --- |
| Hypertension | 35% | 58% | 33% | 36% | 0.82 |
| Cardiometabolic | 45% | 32% | 43% | 36% | 0.37 |
| Psychiatric | 5% | 5% | 0% | 7% | 0.55 |
| Thyroid | 25% | 16% | 14% | 29% | 0.67 |
| Estrogen replacement | 0% | 0% | 0% | 14% | 0.07 |

Note:

p-values are given for generalized linear models.

Psychiatric medications refer to those prescribed for depression, anxiety disorders, or restless leg syndrome

Cardiometabolic medications refer to those prescribed for hyperlipidemia and diabetes

Table S2. Linear mixed effects model of cognitive function during dual task for individuals who completed the 6 week test

|  | Sum Sq | Mean Sq | NumDF | DenDF | F value | Pr(>F) |
| --- | --- | --- | --- | --- | --- | --- |
| Group | 68.58 | 22.86 | 3 | 76.041 | 1.6628 | 0.18211 |
| Time | 531.95 | 265.973 | 2 | 114.614 | 19.3464 | 5.76E-08 |
| Group:Time | 190.25 | 31.709 | 6 | 114.572 | 2.3065 | 0.03867 |

Table S3. Post-hoc contrasts for Linear mixed effects model of cognitive function during dual task for individuals who completed the 6 week test

|  | Estimate | Std. Error | z value | Pr(>\|z\|) |
| --- | --- | --- | --- | --- |
| baseline.1 - outcome06.1==0 == 0 | -3.8143 | 1.3498 | -2.826 | 0.0189 |
| baseline.1 - outcome12.1==0 == 0 | -8.3546 | 1.5073 | -5.543 | 3.57E-07 |
| outcome06.1 - outcome12.1==0 == 0 | -4.5403 | 1.5165 | -2.994 | 0.0165 |
| baseline.2 - outcome06.2==0 == 0 | -2.815 | 1.3143 | -2.142 | 0.0552 |
| baseline.2 - outcome12.2==0 == 0 | -3.0532 | 1.3143 | -2.323 | 0.0484 |
| outcome06.2 - outcome12.2==0 == 0 | -0.2382 | 1.3195 | -0.181 | 0.8567 |
| baseline.3 - outcome06.3==0 == 0 | 0.6365 | 1.5049 | 0.423 | 0.7334 |
| baseline.3 - outcome12.3==0 == 0 | -2.7293 | 1.5502 | -1.761 | 0.1174 |
| outcome06.3 - outcome12.3==0 == 0 | -3.3658 | 1.5541 | -2.166 | 0.0552 |
| baseline.4 - outcome06.4==0 == 0 | -1.9988 | 1.4356 | -1.392 | 0.2185 |
| baseline.4 - outcome12.4==0 == 0 | -3.931 | 1.6365 | -2.402 | 0.0484 |
| outcome06.4 - outcome12.4==0 == 0 | -1.9323 | 1.6526 | -1.169 | 0.2908 |

Note: Groupings are – 1:EXCOG, 2:EX, 3:CON, 4:COG. For example, baseline.1 refers to baseline values for the EXCOG group.

Table S4. Linear mixed effects model of dual task cost on stride length

|  | Sum Sq | Mean Sq | NumDF | DenDF | F value | Pr(>F) |
| --- | --- | --- | --- | --- | --- | --- |
| Group | 56.261 | 18.754 | 3 | 57.622 | 2.5809 | 0.062179 |
| Times | 95.106 | 47.553 | 2 | 85.765 | 6.5443 | 0.002264 |
| Group:Times | 22.802 | 3.8 | 6 | 85.53 | 0.523 | 0.7894 |

Table S5. Linear mixed effects model of dual task cost on CV of stride length

|  | Sum Sq | Mean Sq | NumDF | DenDF | F value | Pr(>F) |
| --- | --- | --- | --- | --- | --- | --- |
| Group | 0.10759 | 0.035864 | 3 | 42.288 | 0.7115 | 0.55054 |
| Times | 0.42488 | 0.21244 | 2 | 70.091 | 4.2148 | 0.01869 |
| Group:Times | 0.11417 | 0.019029 | 6 | 69.816 | 0.3775 | 0.89095 |

Table S6. Linear mixed effects model of dual task cost on stride duration

|  | Sum Sq | Mean Sq | NumDF | DenDF | F value | Pr(>F) |
| --- | --- | --- | --- | --- | --- | --- |
| Group | 38.079 | 12.693 | 3 | 50.873 | 0.6303 | 0.598831 |
| Times | 220.297 | 110.148 | 2 | 70.385 | 5.4696 | 0.006195 |
| Group:Times | 65.762 | 10.96 | 6 | 70.243 | 0.5443 | 0.772762 |

Table S7. Linear mixed effects model of dual task cost on CV of stride duration

|  | Sum Sq | Mean Sq | NumDF | DenDF | F value | Pr(>F) |
| --- | --- | --- | --- | --- | --- | --- |
| Group | 1159.9 | 386.64 | 3 | 45.137 | 0.2555 | 0.857 |
| Times | 6010.1 | 3005.07 | 2 | 73.228 | 1.9862 | 0.1445 |
| Group:Times | 8976.2 | 1496.03 | 6 | 72.959 | 0.9888 | 0.4393 |

Table S8. Linear mixed effects model of dual task cost on stride velocity

|  | Sum Sq | Mean Sq | NumDF | DenDF | F value | Pr(>F) |
| --- | --- | --- | --- | --- | --- | --- |
| Group | 79.67 | 26.557 | 3 | 55.636 | 1.1709 | 0.3291029 |
| Times | 379.72 | 189.861 | 2 | 76.898 | 8.3709 | 0.0005139 |
| Group:Times | 43.36 | 7.227 | 6 | 76.722 | 0.3186 | 0.9253858 |

Table S9. Linear mixed effects model of dual task cost on CV of stride velocity

|  | Sum Sq | Mean Sq | NumDF | DenDF | F value | Pr(>F) |
| --- | --- | --- | --- | --- | --- | --- |
| Group | 713 | 237.68 | 3 | 53.827 | 0.5671 | 0.639103 |
| Times | 5052.6 | 2526.3 | 2 | 83.58 | 6.0273 | 0.003587 |
| Group:Times | 1477.9 | 246.31 | 6 | 83.364 | 0.5876 | 0.739273 |

SUPPLEMENTARY FIGURES
